# Rate and determinants of treatment response to different antiretroviral combination strategies in subjects presenting at HIV-1 diagnosis with advanced disease

**DOI:** 10.1186/1471-2334-11-341

**Published:** 2011-12-14

**Authors:** Antonella Esposito, Marco Floridia, Gabriella d'Ettorre, Daniele Pastori, Alessandra Fantauzzi, Paola Massetti, Giancarlo Ceccarelli, Camilla Ajassa, Vincenzo Vullo, Ivano Mezzaroma

**Affiliations:** 1 Dpt. of Clinical Medicine, "Sapienza" University of Rome, Rome, Italy; 2 Dpt. of Therapeutic Research and Medicines Evaluation, Istituto Superiore di Sanità, Rome, Italy; 3 Dpt. of Public Health and Infectious Diseases, "Sapienza" University of Rome, Rome, Italy; 4 Dpt. of Internal Medicine and Medical Specialties, "Sapienza" University of Rome, Rome, Italy

## Abstract

**Background:**

The optimal therapeutic strategies for patients presenting with advanced disease at HIV-1 diagnosis are as yet incompletely defined.

**Methods:**

All patients presenting at two outpatient clinics in 2000-2009 with an AIDS-defining clinical condition or a CD4+ T cell count < 200/μL at HIV-1 diagnosis were analyzed for the presence of combined immunovirological response, defined by the concomitant presence of an absolute number of CD4+ T cells > 200 cells/μL and a plasma HIV-1 RNA copy number < 50/mL after 12 months of HAART.

**Results:**

Among 102 evaluable patients, first-line regimens were protease inhibitors [PI]-based in 78 cases (77%) and efavirenz-based in 24 cases (23%). The overall response rate was 65% (95% CI: 55-74), with no differences by gender, age, nationality, route of transmission, hepatitis virus coinfections, presence of AIDS-defining clinical events, baseline HIV-1 viral load, or type of regimen (response rates with PI-based and efavirenz-based therapy: 63% and 71%, respectively, p = 0.474). Response rate was significantly better with higher baseline CD4+ T cell counts (78% with CD4+ ≥ 100/μL, compared to 50% with CD4+ < 100/μL; odds ratio: 3.5; 95% CI: 1.49-8.23, p = 0.003). Median time on first-line antiretroviral therapy was 24 months (interquartile range: 12-48). Switch to a second line treatment occurred in 57% of patients, mainly for simplification (57%), and was significantly more common with PI-based regimens [adjusted hazard ratios (AHR) with respect to efavirenz-based regimens: 3.88 for unboosted PIs (95% CI: 1.40-10.7, p = 0.009) and 4.21 for ritonavir-boosted PI (95%CI 1.7-10.4, p = 0.002)] and in older subjects (≥ 50 years) (AHR: 1.83; 95% CI: 1.02-3.31, p = 0.044). Overall mortality was low (3% after a median follow up of 48 months).

**Conclusions:**

Our data indicate that a favorable immunovirological response is possible in the majority of naive patients presenting at HIV-1 diagnosis with AIDS or low CD4+ T cell counts, and confirm that starting HAART with a more compromised immune system may be associated with a delayed and sometimes partial immune recovery. Simpler regimens may be preferable in this particular population.

## Background

In Human Immunodeficiency Virus type 1 (HIV-1) infection, the introduction of highly active antiretroviral therapy (HAART) has determined a marked decrease in HIV-1 related morbidity and mortality [1]. However, some important questions remain open about the best therapeutic strategies for particular groups of HIV-1 infected patients, such as subjects who are diagnosed with HIV-1 infection when the disease has already progressed to a severe level of immune deterioration. Such subjects are commonly defined as "AIDS presenters" when the diagnosis of HIV-1 infection is concurrent with an AIDS-defining opportunistic disease (Group C according to the Centers for Disease Control classification system) [2] or with a number of CD4+ T cells near or below 200/μL. Similarly, the definition of "late presenters" has been recently proposed for those persons with a clinical AIDS-defining condition or a number of CD4+ T cells below 350/μL at diagnosis [3]. A late diagnosis is usually more common among individuals belonging to categories considered at lower risk, such as non promiscuous heterosexual or aged individuals, or among individuals belonging to "marginalized" categories, such as immigrants, who have less frequent access to health services or medical tests. Conversely, a late diagnosis is less frequent among people who perceive themselves as at risk, such as injecting drug users, or among men who have sex with men (MSM), who may be diagnosed with HIV-1 at an earlier stage [4-8]. It is estimated that in industrialized countries a quarter of patients with HIV-1 disease are not aware of their infective status. Many of these individuals come to a late diagnosis, often when they are hospitalized for the occurrence of an opportunistic disease. Several studies show that the proportion of "late presenters" among new diagnoses ranges between 15 and 43% [6,9]. Late presenters have a higher short-term mortality, higher morbidity, and may transmit HIV-1 infection more commonly, either because they have a low perception of risk and therefore do not implement preventive behaviors, or because they have high HIV-1 RNA levels in plasma [10]. In addition, after starting HAART, late presenter patients have an increased risk of developing the immune reconstitution inflammatory syndrome (IRIS), or may show a suboptimal immune recovery, with persistently low levels of CD4+ T cells despite full viral suppression [11-15].

Despite the general consistency of all HIV-1 treatment guidelines in recommending antiretroviral therapy in the presence of clinical and/or laboratory situations indicating immune deterioration [16,17], there are several problems associated with antiretroviral therapy in HIV-1 late presenters regarding the time of its initiation and the choice of first-line antiretroviral drugs. The presence of opportunistic infections significantly complicates their therapeutic management, because the concomitant treatment of HIV-1 and opportunistic conditions may determine negative pharmacokinetic and pharmacodynamic interactions, or reduce levels of therapeutic adherence because of the higher amount of tablets and doses needed [18]. A commonly endorsed approach is to treat the concomitant opportunistic disease before starting antiretroviral therapy [19], but antiretroviral treatment guidelines do not provide more precise indications according to the severity of immunological deterioration and the presence of opportunistic infections [20]. In clinical practice, when treating patients with significant immune deterioration, antiretroviral regimens based on ritonavir-boosted protease inhibitors (PIs) are frequently preferred to regimens based on non-nucleoside reverse transcriptase inhibitors (NNRTIs). Schemes based on boosted PIs are generally considered more potent, but are also burdened by a higher incidence of metabolic adverse events affecting lipid and glucose metabolism, whereas NNRTI-based regimens have a lower barrier to resistance and may be characterized by pharmacokinetic and pharmacodynamic interactions complicating the initial therapeutic response [21,22]. Overall, it is unclear to which extent PI-based regimens are superior to NNRTI-based regimens in this particular setting [23,24].

The aim of this retrospective study was to identify, in a cohort of HIV-1 infected patients presenting at the diagnosis with an advanced disease, rate and potential determinants of an adequate and sustained immunovirological response, defined by achieving an absolute number of CD4+ T lymphocytes > 200 cells/μL together with plasma HIV-1 RNA levels below the threshold of detection (50 copies/mL) after 12 months of HAART. We considered particularly relevant to evaluate the efficacy of NNRTI-based regimens compared to PI-based regimens with or without ritonavir boost. A secondary objective of our analysis was to evaluate predictors of switching to a second line antiretroviral therapy.

## Patients and methods

### Population

We retrospectively selected, among all the patients tested positive for antibodies to HIV-1 between 2000 and 2009 at the outpatient clinics of the Dpt. of Clinical Medicine and the Dpt. of Infectious and Tropical Diseases, "Sapienza" University of Rome, the clinical records of those who presented with a clinical AIDS-defining condition and/or with a CD4+ T cell count below 200/μL. Stage of HIV-1 disease was graded according to the CDC case definition [2]. All patients started HAART [defined by two nucleoside reverse transcriptase inhibitors (NRTIs) plus either a PI (with or without a low-dose ritonavir booster) or a NNRTI] within one month from anti-HIV-1 antibody detection or from the resolution of the acute opportunistic disease that determined the hospitalization. Regimens were selected at the discretion of the treating physician. Information on patient's demographics (date of birth, sex and risk group), clinical events (date of HIV-1 diagnosis, type and date of all AIDS events, and date of death), and laboratory and clinical measurements was taken at the time of HIV-1 diagnosis. Thereafter, patients were followed at three-month intervals with clinical examinations, CD4+ T cell counts and percentages, HIV-1 RNA levels and complete hematological and biochemistry evaluations. Moreover, information on antiretroviral use (dates of starting and stopping antiretroviral drugs, reasons for changing, and side effects) was collected at any scheduled visit. Follow up for time to switch and survival was censored at month 48 of observation.

All patients included in the study gave their informed consent to data collection and the retrospective study was approved by the review board at our Institution.

### Statistical analysis

Quantitative variables were summarized with medians and interquartile ranges. Categorical variables were summarized using proportions. The main outcome measure was represented by immunovirological success, defined by a CD4+ T cell count above 200/μL plus undetectable (< 50 copies/mL) HIV-1 RNA plasma levels at month 12 (interval accepted: 11-13 months) from the start of treatment. Categorical data were compared using the χ^2 ^test, calculating odds ratios with 95%CI by Mantel-Haenszel estimates, or the Fisher test in the presence of at least one cell with < 5 expected cases in contingency tables. Kaplan-Meier survival analysis with log-rank test and Cox regression were used to analyse time on treatment and to calculate probabilities and hazard ratios for changing first-line treatment. In order to adjust for potential confounders, analyses included multivariable logistic regression analyses with estimates of adjusted odds ratios and 95%CI for immunovirological success, and Cox regression models with estimates of adjusted hazard ratios for switch from first-line treatment. The assumption of proportional hazards was tested by the ln(-ln) transformation procedure. Significance levels were set at 0.05. All the analyses were performed with the SPSS software, Version 17.0 (SPSS Inc., Chicago, IL, US).

## Results

### General characteristics

Based on clinical records, 107 subjects were identified. Of these, 5 were excluded from the analysis because of short follow up (ie < 12 months, without death or disease progression, n: 3), or unconfirmed eligibility criteria (A2 CDC category with CD4 > 200/μL, n: 2), for a total of 102 evaluable patients. The proportion of late presenters (AIDS-defining clinical event or CD4 < 200/μL) across the entire study period (2000-2009) was 28.7%. The main patients' characteristics at start of treatment, as well as the proportion of patients with immunovirological success and with switch of treatment are shown in Table 1. Thirty-eight (37%) were female; median age was 44 (interquartile range [IQR]: 37.5-50), and most of the patients (81%) were of Italian nationality. The most common route of transmission was represented by unprotected sexual intercourses in heterosexual men or women (72%) or homo-bisexual men (23%), followed by injection drug use (3%) and exposure to infected blood through transfusion or use of blood components (2%). Forty-three patients (42%) were tested for HIV-1 due to the occurrence of an opportunistic disease: 16 (37%) for *Pneumocystis jirovecii *pneumonia, 7 (16%) for esophageal candidiasis, 6 (14%) for neurotoxoplasmosis, 4 (9%) for tuberculosis, 4 (9%) for a wasting syndrome, 3 (7%) for disseminated cryptococcosis, 2 (5%) for Kaposi's sarcoma, and 1 (2%) for cytomegalovirus retinitis. At the time of diagnosis 36 patients (35%) were in CDC stage A3, 23 (23%) in B3, 1 (1%) in C2 and 42 (41%) in C3. Coinfection with hepatitis viruses was present in 9 patients (9%). The median values of CD4+ T cells and plasma HIV-1 RNA at diagnosis were 104.5/μL (IQR: 48-160), and 5.26 log_10_/mL (IQR: 4.99-5.58).

**Table 1:** Immunovirological response and treatment switch by baseline characteristics

	n with immunovirological success (%)	immunovirological success: odds ratio (95%CI)	p value	n (%) with treatment switch within 48 months	estimated probability of switch within 48 months (95% CI)	Hazard ratio (95%CI) for switch within 48 months	p value
**Gender**							
males (n: 64, 63%)	41/64 (64)	0.93 (0.40-2.15)	0.860	39/64 (61)	0.67 (0.54-0.80)	1.23 (0.63-2.42)	0.533
females (n: 38, 37%)	25/38 (66)			19/38 (50)	0.56 (0.38-0.74)		

**Age **(years)							
median (IQR): 44 (37.5-50)							
≥ 50 (n: 27, 26%)	20/27 (74)	1.80 (0.68-4.79)	0.235	19/27 (70)	0.72 (0.54-0.90)	1.73 (0.93-3.22)	0.085
< 50 (n: 75, 74%)	46/75 (61)			39/75 (52)	0.61 (0.48-0.74)		

**Nationality**							
Italy (n: 83, 81%)	54/83 (65)	1.09 (0.39-3.06)	0.876	50/83 (60)	0.67 (0.56-0.78)	1.61 (0.70-3.69)	0.262
Other countries (n: 19, 19%)	12/19 (63)			8/19 (42)	0.52 (0.25-0.79)		

**Transmission**							
Heterosexuals (n: 73, 72%)	46/73 (63)	reference category		42/73 (57)	0.66 (0.54-0.78)	reference category	
men who have sex with men (n: 24, 23%)	17/24 (71)	1.42 (0.52-3.88)	0.487	14/24 (58)	0.73 (0.52-0.94)	0.90 (0.45-1.80)	0.768
IVDUs/infected blood (n: 5, 5%)	3/5 (60)	0.88 (0.14-5.61)	0.893	2/5 (40)	0.60 (0.16-1.00)	0.57 (0.13-2.55)	0.460

**HIV clinical stage**							
AIDS (n: 43, 42%)	27/43 (63)	0.86 (0.38-1.97)	0.730	22/43 (51)	0.60 (0.43-0.77)	0.76 (0.43-1.34)	0.335
non-AIDS (n: 59, 58%)	39/59 (66)			36/59 (61)	0.68 (0.54-0.82)		

**Hepatitis virus coinfections**							
Yes (n: 9, 9%) *	5/9 (56)	0.66 (0.16-2.61)	0.550	6/9 (67)	0.72 (0.40-1.00)	1.60 (0.65-3.96)	0.308
No (n: 93, 91%)	61/93 (66)			52/93 (56)	0.63 (0.52-0.74)		

**Baseline CD4+ T cells/μL**							
median (IQR): 104.5 (48-160)							
< 100/μL (n: 48, 47%)	24/48 (50)	3.50 (1.49-8.23)	***0.003***	27/48 (56)	0.68 (0.52-0.84)	0.97 (0.55-1.70)	0.919
≥ 100/μL (n: 54, 53%)	42/54 (78)			31/54 (57)	0.61 (0.47-0.75)		

**Baseline HIV-RNA (copies/mL, log_10_)**							
median (IQR): 5.26 (4.99-5.58)							
< 5 log_10_/mL (n: 26, 25%)	19/26 (73)	0.60 (0.22-1.59)	0.304	12/26 (46)	0.54 (0.32-0.76)	0.88 (0.44-1.76)	0.717
≥ 5 log_10_/mL (n: 76, 75%	47/76 (62)			46/76 (60)	0.67 (0.55-0.79)		

**First-line therapy**							
PIs ** (n: 78, 77%)	49/78 (63)	1.44 (0.53-3.88)	0.474	52/78 (67)	0.75 (0.64-0.86)	4.41 (1.81-10.76)	***0.001***
NNRTIs (efavirenz: n: 24, 23%)	17/24 (71)			6/24 (25)	0.27 (0.07-0.47)		

*HBV: 4 (4%); HCV: 3 (3%); HBV+HCV: 2 (2%).

** Unboosted PIs: 16 (indinavir: 7, nelfinavir: 8, saquinavir: 1); Boosted PIs: 62 (indinavir 2, lopinavir 60)

First-line regimens were PI-based in 78 cases (77%) and NNRTI-based in the remaining 24 cases (23%), and included in all cases two NRTIs as backbone. Among subjects on PI-based regimens, 16 were taking an unboosted PI: (indinavir 7, nelfinavir 8, and saquinavir 1), and 62 a ritonavir-boosted PI (lopinavir 60, and indinavir 2). The only NNRTI used was efavirenz (24 patients). The main characteristics of patients according to treatment (PIs or efavirenz) are shown in Table 2. No significant differences were observed between these two groups.

**Table 2:** Main characteristics of patients by treatment group (efavirenz vs. protease inhibitors)

	On efavirenz (n: 24)	On protease inhibitors (n: 78)	p value
**Gender**			
male (n: 64, 63%)	14/24, 58%	50/78, 64%	0.609
female (n: 38, 37%)	10/24, 42%	28/78, 36%	

**Age **(years)			
≥ 50 (n: 27, 26%)	9/24, 37%	18/78, 23%	0.161
< 50 (n: 75, 74%)	15/24, 62%	60/78, 77%	

**Nationality**			
Italy (n: 83, 81%)	19/24, 79%	64/78, 82%	0.751
Other countries (n: 19, 19%)	5/24, 21%	14/78, 18%	

**Transmission**			
Heterosexual (n: 73, 72%)	19/24, 79%	54/78, 69%	0.269
men who have sex with men (n: 24, 23%)	3/24, 12%	21/78, 27%	
IVDU/infected blood (n: 5, 5%)	2/24, 8%	3/78, 4%	

**HIV clinical stage**			
AIDS (n: 43, 42%)	8/24, 33%	35/78, 45%	0.317
non-AIDS (n: 59, 58%)	16/24, 67%	43/78, 55%	

**Hepatitis virus coinfections**			
No (n: 93, 91%)	20/24, 83%	73/78, 94%	0.121
Yes (n: 9, 9%)	4/24, 17%	5/78, 6%	

**Baseline CD4+ T cells/μL**			
< 100/μL (n: 48, 47%)	8/24, 33%	40/78, 51%	0.123
≥ 100/μL (n: 54, 53%)	16/24, 67%	38/78, 49%	

**Baseline HIV-RNA (copies/mL, log_10_)**			
< 5 log_10_/mL (n: 26, 25%)	8/24, 33%	18/78, 23%	0.313
≥ 5 log_10_/mL (n: 76, 74%	16/24, 67%	60/78, 77%	

### Immunovirological response

The primary end point (combined immunovirological success) was achieved in 66 out of 102 patients, for an overall response rate of 65% (95% CI: 55-74). No difference in response rate was observed by gender, age (< 50 vs. ≥ 50 years), nationality (Italian vs. non-Italian), route of transmission, hepatitis virus coinfections, presence of AIDS-defining events at diagnosis, or baseline HIV-1 viral load (< 5 log_10 _vs. > 5 log_10 _copies/mL) (Table 1).

Response rate was however significantly different by baseline CD4+ T cell levels. The proportion of patients who had both undetectable (< 50 copies/mL) viral load and CD4+ T cell counts above 200/μL at 12 months was significantly higher among patients with a baseline CD4+ T cell count > 100/μL (42/54, 78%) compared to patients with CD4+ counts below this threshold (24/48, 50%; odds ratio: 3.50; 95% CI: 1.49-8.23, p = 0.003). No significant differences were also observed between patients on PIs and on efavirenz: after 12 months, 63% of patients treated with a PI-based HAART (49/78) and 71% of patients treated with efavirenz (17/24) achieved the immunovirological primary response measure (p = 0.47) (Table 1). The groups were also similar in mean changes from baseline for CD4+ T cells and HIV-1 RNA levels. The 12-month mean increase in CD4+ T cells was 176/μL with PIs and 169/μL with efavirenz (p = 0.80), whereas the mean reduction in viral load from baseline was 3.34 log_10_/copies/mL in the PIs group compared to 3.35 log_10 _in the efavirenz group (p = 0.94).

Finally, among patients taking PIs there was no significant difference between subjects treated with and without a ritonavir booster. The average increase from baseline in CD4+ T cells was 171/μL in the boosted PIs group vs. 194/μL in the unboosted PIs group (p = 0.52), with a viral load reduction of 3.41 and 3.08 log_10_, respectively (p = 0.15).

**Figure 1:**
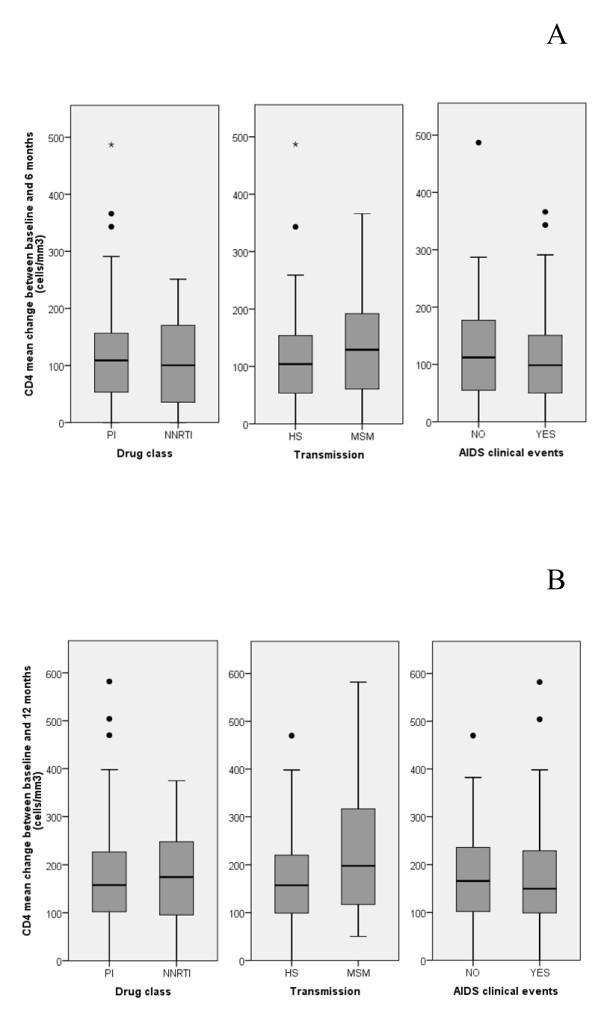
**Changes of CD4+ T cells during time**. CD4+ T cell mean absolute increase from baseline at 6 (A) and 12 months (B) by drug regimen (PIs- *vs*. NNRTIs- based), route of transmission, and clinical AIDS status at enrollment. **Note: **PIs: protease inhibitors; NNRTIs: non-nucleoside reverse transcriptase inhibitors; HS: heterosexual; MSM: men who have sex with men; IVDU: intravenous drug users.

The absolute increase in CD4+ T cells after 6 and 12 months of HAART also showed no significant differences according to the baseline regimen (PIs vs. efavirenz) or the presence of an AIDS-defining condition at baseline (Figure 1). With respect to the route of HIV-1 transmission, however, a significantly higher mean (± SD) increase from baseline in CD4+ T cells was observed among MSM/bisexual men compared to heterosexual men and women (231 ± 136 vs. 163 ± 100 cells/μL, p = 0.01) after 12 months, despite no significant baseline differences between the two groups in clinical stage (44% of AIDS-presenters in heterosexuals vs. 29% among MSM, p = 0.1) and age (44.1 years in heterosexuals vs. 43.2 in MSM, p = 0.73). In order to adjust for possible clinical differences (i.e. different proportion of patients with AIDS between groups) and for the non-randomized attribution of treatment, a multivariable logistic regression model that used immunovirological success as dependent outcome and age (≥ 50 vs. < 50), type of regimen (PI-based vs. NNRTI-based), route of transmission, AIDS status, baseline CD4+ T cells (≥ 100/μL vs. < 100 μL) and HIV-1 RNA levels (≥ 5 log_10 _vs. < 5 log_10 _copies/mL) as covariates was constructed. The results of this analysis substantially confirmed the predictive role of a baseline CD4+ T cell value ≥ 100/μL on immunovirological success, with an adjusted odds ratio of 2.93 compared to CD4+ T cells < 100/μL (95%CI 1.16-7.39, p = 0.023), with no significant associations for the other variables (Table 3).

**Table 3:** Multivariable analyses for the main study outcomes (immunovirological success and switch of treatment)

	Logistic regression model Outcome: immunovirological success		Cox regression model Outcome: switch from first-line therapy	
	**Adjusted odds ratio with 95%CI**	**p value**	**Adjusted hazard ratio with 95%CI**	**p value**

**Age **(years)				
≥ 50	2.03 (0.66-6.23)	0.214	1.83 (1.02-3.31)	***0.044***
< 50	reference category		reference category	

**Transmission**				
Heterosexual	reference category		reference category	
men who have sex with men	1.54 (0.52-4.56)	0.433	0.87 (0.47-1.60)	0.653
IVDU/infected blood	0.72 (0.09-5.38)	0.746	0.60 (0.14-2.66)	0.503

**HIV clinical stage**				
AIDS	reference category		reference category	
non-AIDS	0.70 (0.26-1.85)	0.475	1.13 (0.86-1.50)	0.382

**Baseline CD4+ (T cells/μL)**				
< 100/μL	reference category		reference category	
≥ 100/μL	2.93 (1.16-7.39)	***0.023***	1.00 (0.75-1.33)	0.997

**Baseline HIV-RNA (copies/mL, log_10_)**				
≥ 5 log_10_/mL	reference category		reference category	
< 5 log_10_/mL	1.73 (0.56-5.38)	0.344	0.92 (0.65-1.29)	0.628

**First-line therapy**				
NNRTIs	reference category		reference category	
Unboosted PIs *	1.25 (0.25-6.25)	0.788	3.88 (1.40-10.7)	***0.009***
Boosted PIs **	0.78 (0.25-2.41)	0.663	4.21 (1.70-10.4)	***0.002***

* Unboosted PIs: 16 (indinavir: 7, nelfinavir: 8, saquinavir: 1);

** Boosted PIs: 62 (indinavir 2, lopinavir 60)

### Switch to a second line treatment and survival

**Figure 2:**
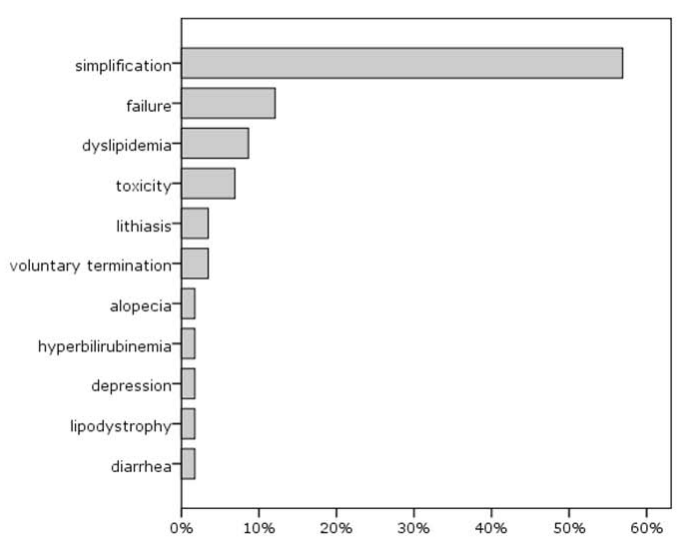
**Reasons for changing first-line regimen (%)**.

First-line antiretroviral therapy had a median duration of 24 months (IQR: 12-48), and switch to a second line treatment occurred in 58 out of 102 patients (57%), mainly for therapy simplification (33/58, 57%). Reasons for change are reported in Figure 2. Switch was not significantly associated with gender, nationality, route of transmission, hepatitis virus coinfections, presence of AIDS-defining conditions, baseline HIV-1 RNA levels or CD4+ T cell counts (Table 1). A not significant trend for a higher risk of change among older patients was observed, whereas the inclusion of a PI (with or without ritonavir booster) in the first regimen was significantly associated with a switch to a second-line treatment: 67% of patients receiving a PI-based first-line regimen (52/78) switched to a second-line therapy, compared to 25% (6/24) of those receiving an efavirenz-based first-line regimen (Table 1). In a Kaplan-Meier analysis, mean time on first-line treatment was significantly longer with NNRTIs (40.6 months) than with PIs, either with low-dose ritonavir (28.3 months, p = 0.003) or without ritonavir booster (30.0 months, p = 0.016, log-rank test). The unadjusted hazard ratio for switching from any PI-based regimen (compared to efavirenz-based regimens as reference) was 3.65 (95% CI: 1.56-8.53, p = 0.003). In a multivariable Cox regression model that adjusted for age (≥ 50 vs. < 50), AIDS status, route of transmission, baseline CD4+ T cells (≥ 100/μL vs < 100 μL) and HIV-1 RNA values (≥ 5 log_10 _vs. < 5 log_10 _copies/mL), the AHRs for switch, compared to NNRTI-based regimens, were 4,21 (95%CI: 1.70-10.4, p = 0.002) for ritonavir-boosted PIs regimens and 3.88 (95%CI: 1.40-10.7, p = 0.009) for unboosted PIs regimens (Table 3). Older age at baseline (≥ 50 years) showed an association with subsequent switch of borderline statistical significance (HR: 0.54, 95% CI: 0.30-0.98, p = 0.044).

Overall mortality was low, and no patient died during the first year of observation; subsequently one patient with non-Hodgkin lymphoma and other two with severe opportunistic infections died between 20 and 48 months, for an overall mortality of 3% during an average follow up of 45 months.

## Discussion

Although guidelines for the diagnosis and treatment of HIV-1 infection recommend starting therapy when number of CD4+ T cells is < 500-350/μL [16,17], it is not uncommon in clinical settings to observe a later initiation of HAART in patients who present with advanced HIV-1 disease. The proportion of "late presenters/AIDS presenters" in our series of new HIV-1 diagnoses (28.7%) is fully consistent with the data from the large Italian cohort of antiretroviral-naive patients (Icona), where 29% of 968 patients enrolled between 1997 and 2000 were first tested for anti-HIV-1 antibodies after the onset of an AIDS-defining condition and/or with a number of CD4+ T lymphocytes < 200/μL. In our group of advanced naive patients, median age was 44 years (with 74% younger than 50 years at diagnosis), slightly lower compared to other studies [6]. The route of transmission distribution, characterized by about 70% of heterosexual contacts, is consistent with recent literature data and with the recent trends in the epidemic, increasingly affecting heterosexuals [25]. These findings may also be related to a different risk perception in different groups, with MSM and injecting drug users probably more likely to be aware of the risk and carry out more frequent testing. Finally, the predominant acquisition of HIV-1 infection by unprotected sexual intercourses may explain the low number of patients with hepatitis virus coinfections in our series of recent diagnoses. With regard to nationality, the vast majority of late presenter patients were of Italian nationality (81%). This represents a difference with other reports, that have indicated an association of the "advanced naive" status with foreign nationality, social marginalization, and limited access to clinical investigations [4-8]. Summarizing our findings, the patient defined as "advanced naive" in our context is mainly a person below 50 years, of Italian nationality, who acquired the infection through heterosexual intercourses.

Considering the combined immunovirological outcome, observed response rate in our series was 65%, with a low mortality (3%) during follow up. Although the proportion of responder subjects may appear slightly lower compared to other studies performed in naive patients [26,27], it is important to note that we considered as responders only those individuals who reached both an undetectable viral load (HIV-1 RNA level < 50 copies/mL) and a CD4+ T cell count above 200/μL after one year of HAART. This strict definition, together with the immunological characteristics of our population and the antiretroviral combinations used, may explain the differences in response rate observed when compared with other studies.

As previously reported, an important goal of the present analysis was to identify predictors of immunovirological success. This outcome was not associated with age, nationality, route of transmission, clinical stage of HIV-1 disease, and HIV-1 viral load at baseline. Conversely, CD4+ T cell count at entry represented a significant predictor: patients starting HAART with CD4+ T cells > 100/μL had a 3 times higher probability to reach an immunovirological response at 12 months. This is consistent with other data showing that starting HAART in a poor immune condition is associated with a delayed, and sometimes partial, immune recovery and suggests that a discordant response to HAART (defined by the immunological non-responder condition) may be more frequent in patients starting therapy with a more severe degree of immune deterioration [28].

Regarding type of first-line treatment, in terms of treatment response our data showed that PI-based regimens were not superior to those based on efavirenz. These data are consistent with the recent ACTG 5142 study, that showed among treatment-naive patients no significant difference in immune recovery after 48 weeks between lopinavir/ritonavir and efavirenz as initial therapy [22], and with other studies in patients with CD4+ T lymphocytes < 100/μL, all demonstrating that efavirenz-induced immune reconstitution was not inferior to that induced by boosted PIs [21,23,24]. In our study the two treatment approaches also displayed similar virological suppression, with no statistically significant differences in the average reduction of plasma viral load after 12 months of treatment. However, the third drug chosen for first-line therapy was an important determinant for the switch to a second-line treatment: 67% of patients receiving a PI-based therapy changed their treatment, compared to 25% alone of patients receiving an efavirenz-based first-line, with a four-fold higher risk of switching for PI-based compared to efavirenz-based regimens. It is important to note that switch took place in more than half of the cases (57%) with the aim to simplify treatment. In fact, PI-based regimens could be more cumbersome to follow, due to a higher number of pills and to dietary restrictions linked to drug assumption. It is therefore likely that such regimens were more frequently changed because of patient request and in order to avoid compromising adherence and therapeutic effectiveness. Our data also suggest that older age might be associated with a higher risk of changing first-line treatment. Even if we were unable to assess the reasons underlying this association, the increased occurrence of comorbidities and concomitant treatments in older patients might facilitate a change of treatment in order to maintain adherence to complex therapeutic schedules and prevent undesired adverse events.

An important point emerged from our study is represented by the differences in immune recovery after one year of therapy in subjects with different routes of transmission. In particular, the 12 month CD4+ T cell recovery seems to be significantly higher in MSM than in heterosexuals. We evaluated potential confounding factors, such as age or AIDS diagnosis, without detecting significant differences. It could be hypothesized that the two groups differ in terms of adherence to the therapeutic prescriptions. It could be particularly relevant to explore this issue promoting studies on adherence to antiretroviral therapy in late presenters, in order to evaluate this hypothesis and simplify as much as possible the treatment in patients identified as less compliant with drug prescriptions. Selecting a regimen that ensures maximum adherence to get as soon as possible an adequate immune recovery is particularly important in these advanced patients, who may have more compromised clinical and/or immunological conditions.

Our study has some limitations: a limited sample size, that may have reduced the power to detect differences between groups, a non-randomized assignation of treatment, and the lack of adherence measurements. The findings related to treatment should therefore be taken cautiously. Moreover, we cannot extend the validity of these observations to other NNRTIs, such as nevirapine and etravirine. Nonetheless, our data suggest that based on a background of similar immunovirological response, efavirenz-based regimens could have an advantage over PI-based regimens because of their simpler administration characteristics, that might promote better adherence in a sustainable long-term approach. Finally, in terms of possible therapeutic choices, it is important to consider in these particular patients the potential use of other recently available treatment options [27,29] represented by new classes of antiretroviral drugs (i.e., integrase inhibitors and CCR5 inhibitors) or new NNRTIs (i.e., rilpivirine), characterized by particularly favourable dynamics of viral load reductions and CD4+ T cell recovery, and probably accompanied by less marked metabolic effects.

## Conclusions

Despite increasing progress in treatment of individuals with HIV-1 disease, a late diagnosis remains the main factor contributing to the occurrence of new AIDS cases and persistence of a high mortality, and represents a major obstacle to an effective prevention of infection spread in Western countries. The implementation of information campaigns remains essential to promote the adoption of effective preventive behaviours and to achieve an earlier and more diffuse HIV-1 testing. Our data indicate the possibility to reach a favorable immunovirological response in the majority of naive patients presenting at HIV-1 diagnosis with AIDS or low CD4+ T cells, and confirm that starting HAART with a more severely compromised immune system may be associated with a delayed, and sometimes partial, immune recovery. Furthermore, these observations strongly suggest that simpler regimens may be a preferable therapeutic option in this particular population due to their better sustainability in a long-term prospective.

## Competing interests

The authors declare that they have no competing interests.

## Authors' contributions

All authors 1) have made substantial contributions to conception and design, or acquisition of data, or analysis and interpretation of data; 2) have been involved in drafting the manuscript or revising it critically for important intellectual content; and 3) have given final approval of the version to be submitted.

## Pre-publication history

The pre-publication history for this paper can be accessed here:

http://www.biomedcentral.com/1471-2334/11/341/prepub
